# Society Meetings

**Published:** 1914-05

**Authors:** 


### SOCIETY MEETINGS

Brief reports and announcements of meetings of societies of Western New York, and those of general scope, are requested from Secretaries. Copy should be on hand the fifteenth of the month. Full transactions will be published at cost of composition.

### Medical Society of the County of Erie.

The regular meeting of the Medical Society of the County of Erie was held at the Hotel Touraine April 20th, 1914.

This meeting was preceded by a subscription dinner to meet Dr. William Francis Campbell, president of the Medical Society of the State of New York. About 125 members attended. President Woodruff acted as toastmaster.

In the course of his remarks, he expressed the hope that the Western end of the State would be honored as is now the Eastern end. This caused a spontaneous outburst for Dr. Grover W. Wende, the society's candidate for president of the State Society.

Dr. Campbell was accompanied by Dr. James W Flemming of Brooklyn, who in the course of his remarks, encouraged his audience by saying that Brooklyn was with Buffalo.

Dr. W. T. Shanahan, of Sonyea, president of the 7th District Branch, brought greetings and encouragement from his district. The president then called upon Secretary Gram to give a condensed account of the activities of the Medical Society of the County of Erie during the past century.

Other speakers were Dr. Edith R. Hatch, Dr. N. Victoria Chappell, Dr. Arthur G. Bennett and Dr. Thomas H. McKee, of Buffalo, and Dr. S. E. Page of East Bethany, N. Y., secretary of the Genesee County Medical Society.

At 8.30 o'clock, the regular meeting of the County Society was called to order by President John V. Woodruff.

The minutes of the previous meeting, held February 16th, 1914, were adopted without reading, having been published. (See March issue).

The secretary then read the minutes of the Council meetings of March 2nd and April 6th, 1914, both of which were adopted as read.

The following amendments to the by-laws, which had been offered at the previous meeting, and published with the notice of the meeting of April 20th, 1914, as required, were then read and adopted:

To amend Chapter 2, of the By-laws, by introducing a section to be known as section 13, which should read:

Section 13. **Retired members.**—Members in good standing who are seventy years of age or over may, by a majority vote of the Society present and voting at any annual meeting, become retired members subject to the approval of the State Society as defined in its by-laws, Chapter 1, section 2. Applicants for retired membership must be approved and endorsed by the council and the application must be sent to the secretary of the State Society in time for presentation at the first meeting of the house of delegates. Retired members shall be entitled to the privilege of attending and addressing the meetings of the Society, but shall not be accorded other rights or privileges of membership, nor be subject to assessments.

Amend Chapter 7, section 1 by introducing a new line to read—''4. Economics'' after the line ''3. Membership.''

Amend Chapter 7 by the introduction of a new section to be known as section 6 which shall read: **Committee on Economics.**—The Committee on Economics shall consist of three members including the chairman. It shall be the duty of this committee to investigate and to report to the Council with recommendations all questions of an economic nature not properly the duty of other committees, or those referred to it by the Society such as political, financial and educational relations with the government, with other professions, with the laiety and with members of the medical profession and of the Society. It shall have power to apoint temporary subcommittees approved by the Council. It shall co-operate with like committees of the State and National Societies. Two members shall constitute a quorum.

Dr. Albert T. Lytle then presented a comprehensive paper on ''Contract Practise, an Economic Study.''

Dr. William Francis Campbell was then introduced by the president, and entertained the Society with a scholarly address embodying his observations made during the past year in his visits to the various county and district societies in an official capacity .

Dr. Lytle's paper was then thoroughly discussed and various views were presented.

A reception and buffet lunch followed the meeting.

FRANKLIN C. GRAM, M. D.,

Secretary.

---

**The Buffalo Academy of Medicine** has held the following meetings:

Stated Meeting of Academy, March 24 ,at which a report was made by the special committee to investigate building sites. The Section on Pathology furnished the scietific program: Some Tropical Granulomata, Dr. Richard P. Strong of Harvard University.

Section on Surgery, March 31: Sclero-corneal Trephining in the Operative Treatment of Glaucoma, according to the Method of Col. R. H. Elliott, Dr. Lee Masten Francis of Buffalo.

Section on Medicine, April 7: "We have left undone those things which we ought to have done and we have done those things which we ought not to have done." Dr. T. H. McKee of Buffalo; Clinical Experiences with Phenol Therapy, Dr. George R. Critchlow of Buffalo.

Section of Obstetrics and Gynecology, April 14: Program—Five-minute Papers from Practical Experience:

Drainage in Puerperal Fever ............M. Hartwig, M. D.
The Misuse of Forceps ............John V. Woodruff, M. D.
Method of Performing Episeotomy Frank H. Ransom, Jr. M.D.
Occipit Posterior ....................Wm. T. Getman, M.D.
McDonald's Method of Examining for Probable
Time of Labor ..............Geo. R. Critchlow, M. D.
The Fisher Treatment in Eclampsia ..Thomas J .Walsh, M. D.
The Short Cord ........................N. Kavinoky, M. D.
Position of Patient Following Labor ..Irving W. Potter, M. D.
Temperature of the New-born ........Edith R. Hatch, M. D.
Pituitrin, When Not to Use it in Labor..W. W. Britt, M. D.
Estimation of Cervilical Dilitation by
Rectum ...................Herman K. DeGroat, M. D.
All of Buffalo.

Section on Pathology, April 21: Experimental Pneumonia, Dr. M. C. Winternitz of Johns Hopkins.

---

**Rochester Academy of Medicine.** Section IV.—Public Health, including Hygiene, Climatology, Physiology, Pathology, Bacteriology, and Forensic Medicine. The regular meeting of this section was held at the Academy rooms, 355 East Avenue, April 8. The Significance of Albumin in the Urine—1. The chemical tests for Albumin ,Dr. Charles R. Witherspoon. 2. The significance of Albuminuria in the acute infectious diseases, Dr. John R. Williams. 3. The significance of Albuminuria in chronic diseases: (a) Respiratory, Dr. E. G. Whipple; (b) Circulatory, Dr. N. W. Soble; (c) Genito-Urinary, Dr. W. F. Plumley; (d) Gastro-Intestinal, Dr. W. V. Ewers; (e) Nervous, Dr. E. L. Hanes. 4. The significance of Albuminuria to the Surgeon, Dr. C. W. Hennington. 5. The significance of Albuminuria to the Obstetrician, Dr. W. M. Brown. 6. The significance of Albuminuria to the Ophthalmologist, Dr. A. C. Snell. We expect to publish these papers next month.

**Section III.**—Obstetrics, Gynecology, and Pediatrics, met March 11. The Infants Summer Hospital. Some Pediatric Cases, Dr. Joseph Roby.

**The Monroe County Medical Society** holds its annual all-day meeting Tuesday, May 19. The program consists of a clinic by the local members in the various hospitals, then a luncheon, followed by a number of formal papers and addresses during the afternoon. The officers are Dr. A. C. Snell, president, and Dr. C. W. Hennington, secretary.

---

**The Hospital Medical Society of Rochester** held a meeting on Thursday, April 23, at which Dr. F. W. Seymour read upon New Growths of the Mediastinum, with report of two cases. The annual Spring meeting occurs on May 7.

---

The regular meeting of the **Rochester Pathological Society** was held at the Rochester Whist Club, 40 N. Fitzhugh Street, Thursday evening, April 16, 1914. Dr. H. L. Prince gave a paper on Diagnosis and Treatment of Congenital Dislocation of the Hip. W. Douglas Ward, president, Charles L. Hincher, secretary, 270 North Street.

---

**Elmira Academy of Medicine** met April 1st, Modern Opthalmology and Oto-Laryngology, G. M. Case, M. D. Eugenics, F. L. Christian, M. D.

---

Under the auspices of the **New York Branch of the American Pharmaceutical Association** a joint meeting of physicians and pharmacists will be held on the evening of May 18th at 8 o'clock, at the College of Pharmacy Building, 115 West 68th Street, New York City.

The subject will be "Pharmacopoeial Revision." Professor Remington of Philadelphia, Chairman of the Committee of Revision, will lead the discussion.

It is earnestly hoped that the medical profession will be largely represented. The discussion will certainly prove of vital interest to all physicians and pharmacists.

---

The 108th Annual Meeting of the **Medical Society of the State of New York** was held at the Hotel Astor, New York, April 28-30. The registration was nearly 800, with no effort at padding by registration of local members not actually in attendance. The next meeting will be held in Buffalo under the presidency of Dr. Grover W. Wende. A more extensive notice is impossible on account of our date of printing and is undesirable because it would be neither courteous nor an indication of loyalty to the State Society to anticipate or to duplicate the announcement and publication of papers in the Journal of the organization.

